# Personalised 3D Assessment of Trochanteric Soft Tissues Improves HIP Fracture Classification Accuracy

**DOI:** 10.1007/s10439-022-02924-1

**Published:** 2022-02-01

**Authors:** Alessandra Aldieri, Mara Terzini, Alberto L. Audenino, Cristina Bignardi, Margaret Paggiosi, Richard Eastell, Marco Viceconti, Pinaki Bhattacharya

**Affiliations:** 1 PolitoBIOMed Lab, Department of Mechanical and Aerospace Engineering, Politecnico di Torino, Turin, Italy; 2 INSIGNEO Institute for In Silico Medicine, University of Sheffield, Pam Liversidge Building, Sheffield, S1 3JD UK; 3 Academic Unit of Bone Metabolism, University of Sheffield, Sheffield, UK; 4 Department of Industrial Engineering, Alma Mater Studiorum - University of Bologna, Bologna, Italy; 5 Laboratorio di Tecnologia Medica, IRCCS Istituto Ortopedico Rizzoli, Bologna, Italy; 6 Department of Mechanical Engineering, University of Sheffield, Sheffield, UK

**Keywords:** Osteoporosis, Hip fracture risk prediction, Trochanteric soft tissues, Multiscale model

## Abstract

Passive soft tissues surrounding the trochanteric region attenuate fall impact forces and thereby control hip fracture risk. The degree of attenuation is related to Soft Tissue Thickness (STT). STT at the neutral hip impact orientation, estimated using a regression relation in body mass index (BMI), was previously shown to influence the current absolute risk of hip fracture (ARF0) and its fracture classification accuracy. The present study investigates whether fracture classification using ARF0 improves when STT is determined from the subject’s Computed-Tomography (CT) scans (i.e. personalised) in an orientation-specific (i.e. 3D) manner. STT is calculated as the shortest distance along any impact orientation between a semi-automatically segmented femur surface and an automatically segmented soft tissue/air boundary. For any subject, STT along any of the 33 impact orientations analysed always exceeds the value estimated using BMI. Accuracy of fracture classification using ARF0 improves when using personalised 3D STT estimates (AUC = 0.87) instead of the BMI-based STT estimate (AUC = 0.85). The improvement is smaller (AUC = 0.86) when orientation-specificity of CT-based STT is suppressed and is nil when personalisation is suppressed instead. Thus, fracture classification using ARF0 improves when CT is used to personalise STT estimates and improves further when, in addition, the estimates are orientation specific.

**Supplementary Information:**

The online version contains supplementary material available at 10.1007/s10439-022-02924-1.

## Introduction

Fragility fractures of the hip are a major healthcare problem, with over £2 billion being spent annually in their treatment in the UK alone, and with over $10,000 being spent per fracture on average for index hospitalisations globally.^11,32^ Hip fractures can be prevented with more accurate fracture risk prediction. It is well recognised that more accurate prediction of fracture risk can be achieved by higher personalisation in determining an individual’s risk factors.

As large majority of hip fractures result from a fall to the side, much attention has been given to fall frequency, fall severity and bone strength as potential risk factors. One measure of the severity of fall is impact attenuation, i.e. the degree to which the impact force from the floor is attenuated before being transferred to the hip. By some estimates, its variation alone explains over 75% of the population-wide variation in the fall-induced impact forces on the femur.^3^ Active muscular co-contraction around the hip during a fall controls the transfer of forces to the femur to some extent.^17,25,30^ However, it remains challenging to both characterise the variability of muscle activation in a subject across all the falls they can encounter, as well as to model the influence of this activation on the mechanics of impact attenuation. Another contributor to impact force attenuation at the hip is passive attenuation. Here, the most important contributors are flooring materials and clothing (particularly hip protectors^12,23,26^) and trochanteric passive soft tissues.^6,7,16,22,24^ However, only the last of these depends on the subject’s anatomy. Thus, for the objective of increasing the accuracy of fracture risk prediction through greater personalisation, the assessment of the impact force attenuation due to passive trochanteric soft tissues presents an attractive choice.

A test for whether a fracture risk indicator is accurate is whether it classifies existing fractures and non-fractures accurately. A previous study^3^ (henceforth referred to as B18), developed a multiscale model to predict the current absolute risk of hip fracture (ARF0). Amongst all other classifiers considered, the accuracy of ARF0 (quantified as AUC, or the Area Under the Receiver-Operating Characteristic, or ROC, curve) in classifying hip fractures in a cohort of British post-menopausal women was determined to be the highest. The other classifiers that have been applied on this cohort are FRAX, Dual-energy X-ray Absorptiometry based areal Bone Mineral Density (DXA-aBMD) of the femoral neck and minimum femur strength in a sideways fall configuration.^1,21,33^

In B18, ARF0 is defined as the probability of suffering a fracture over a period of a year from clinical presentation, i.e. from the time the CT has been performed. As such, it considers a range of fall scenarios, and computes the probability that at least one of these scenarios will lead to a fracture. Thus, in the ARF0 model developed in B18, several impact orientations of the femur with respect to the ground were considered. Yet, the same model considered the attenuation of impact force due to passive trochanteric soft tissues (henceforth ${\eta }_{\mathrm{ST}}$) in a subject to be impact orientation independent (i.e. a scalar value). ${\eta }_{\mathrm{ST}}$ was estimated based on a regression relationship with Soft-Tissue Thickness (STT) at the point of the greater trochanter (denoted STT0).^24^ STT0 itself was estimated from a regression relationship with the subject’s Body Mass Index (BMI).^4,19^ These regression relationships are well known in the literature but lead to uncertainties in the estimated variables. Indeed, their use as described above leads the standard error in the estimate of subject-specific ${\eta }_{\mathrm{ST}}$ (0.10) to be comparable to its standard deviation across the cohort (0.11). This underscores the potential for higher personalisation of ${\eta }_{\mathrm{ST}}$ by employing a direct measure of STT. The proximal femur CT images which are used to determine bone strength in the ARF0 model provide a ready source for this direct measurement.

In addition, compared to DXA or ultrasound (US),^9,15,27^ proximal femur CT scans can determine the three-dimensional (3D) geometry of trochanteric STT. If the regression relationship^24^ between STT and ${\eta }_{\mathrm{ST}}$ holds for all orientations, then CT scans can be used to determine ${\eta }_{\mathrm{ST}}$ in an orientation-specific manner. To the best of the authors knowledge, such subject- and orientation-specific assessment of trochanteric STT has not been reported in the literature. The present study addresses this lacuna and investigates whether this leads to improved fracture classification accuracy of ARF0.

## Materials and Methods

### Clinical Cohort

Subject-specific proximal femur CT images (slice thickness 0.625 mm; in-plane pixel spacing 0.74 × 0.74 mm^2^) were obtained for 100 postmenopausal British women, comprising 50 fracture cases and 50 control subjects, in a previous retrospective case–control study.^33^ Ethical approval (ethical committee agreement number: 07/H1308/093) for that study was granted by Sheffield Local Research Ethics Committee (North Sheffield REC), and informed written consent from all participants for use of data in further research is held on record. Previous analysis on this cohort was based on 49 fracture and 49 control subjects, due to issues with CT data quality in two subjects.^1,3,21^ Personalised STT measurement (see below) could not be performed for two fracture and two control subjects whose CT data was lost in since the original study.^33^ Hence, the present study is based on the remaining 47 fracture and 47 controls subjects, spanning ages from 56 to 91 years. Summary statistics of this reduced cohort is shown in Table 1.

**Table 1:** Mean (standard deviation in parentheses) of age, weight, height, BMI and T-score for subjects in the fracture and control (or non-fracture) groups and for all subjects in the cohort.

	Age (years)	Weight (kg)	Height (m)	BMI (kg/m^2^)	T-score (–)
Fracture	76 (9.1)	63 (14)	1.6 (0.066)	25 (5.1)	− 2.1 (1.2)
Control	75 (9.0)	65 (12)	1.6 (0.056)	26 (4.4)	− 1.0 (1.0)
All	75 (9.0)	64 (13)	1.6 (0.061)	25 (4.8)	− 1.6 (1.2)

### Existing Models

**Figure 1:**
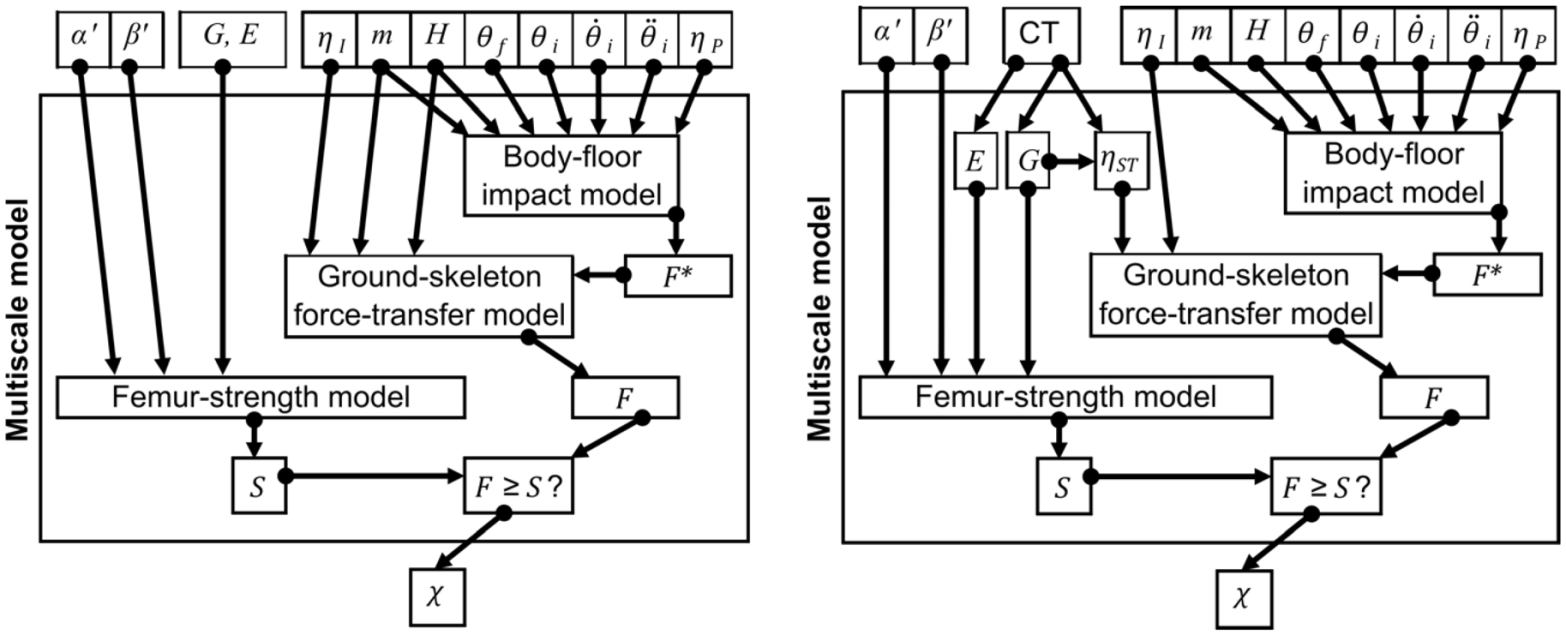
Schematic representations of the ARF0_STT0-DXA-BMI_ model (left, with kind permission from Bhattacharya *et al.*^3^) and the ARF0_STT-CT_ model (right, introduced in the present study). In the ARF0_STT0-DXA-BMI_ model, the femur geometry ($G$) and elastic properties ($E$) are obtained from patient-specific CT images (not shown explicitly above), and ${\eta }_{\mathrm{ST}}$ (part of the ground–skeleton force-transfer model) is estimated from subject’s body mass index (a function of body mass $m$ and height $H$). In the ARF0_STT-CT_ model, patient-specific CT images are used additionally to segment the soft tissue/air boundary and the distance of this surface from the femur surface (encoded in $G$) is used to obtain ${\eta }_{\mathrm{ST}}$.

B18 detailed the original multiscale ARF0 model, along with its verification, uncertainty quantification, validation and sensitivity analysis and that of its component models. This section summarises the original ARF0 model from B18 for reference, while the next section will describe the changes made in the present study. The ARF0 model combined three component models (Fig. 1): an organ scale model (called femur strength model) that yielded bone strength information according to different impact orientation angles; a model at the whole-body scale (called body–floor impact model) that predicted the net impact force; and a model between body and organ scales (called ground–skeleton force-transfer model) that estimated the fraction of the impact force effectively transferred to the skeleton.

**Figure 2:**
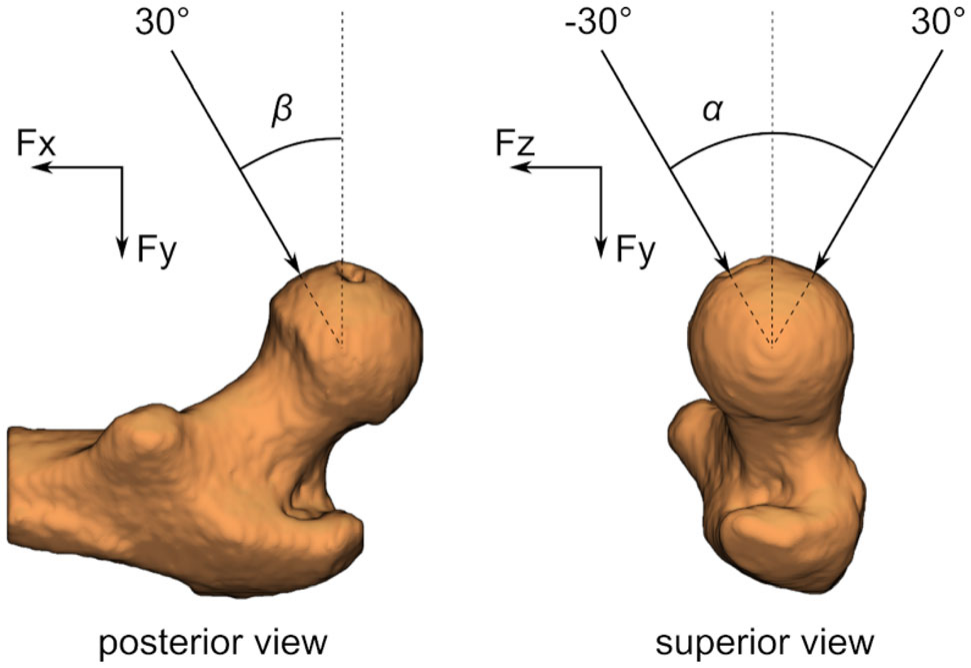
The reference system (adapted with permission from Bhattacharya *et al.*^3^) with the angles $\alpha$ and $\beta$ highlighted. The origin is located at the centre of the femoral head. The anatomical plane passing through the origin, the centres of the femur neck and the diaphysis in the proximal femur contains the longitudinal (Fx) and frontal axes (Fy). The anatomical plane oriented tangential to the femoral condyles and passing through the origin contains the frontal (Fy) and sagittal (Fz) axes.

The femoral strength model is fully described elsewhere.^1,21^ Qasim *et al.*^21^ segmented (ITK-Snap 2.0.0, University of Pennsylvania) one femur for each patient (the contralateral in fractured cases and matched controls) and extracted the three-dimensional bone geometries. They aligned the geometries to the femur anatomical coordinate system (Fig. 2) with its origin located at the femur head centre. The coordinate system was based on anatomical landmarks identified in a corresponding full femur included in an atlas. The segmented femur models were meshed with ten-node tetrahedral elements, with the average element size set to 3 mm following a convergence analysis.^8^ Elastic moduli were mapped onto the meshed bone model (Bonemat, V3) using an empirical relationship.^18^

Altai *et al.*^1^ specified fall impact orientations by angle pairs ($\alpha , \beta$), where $\alpha$ and $\beta$ are, respectively, the rotations about the longitudinal (Fx) and sagittal (Fz) axes in the femur anatomical coordinate system. When $\alpha ={0}^{\circ },\beta ={0}^{\circ }$ (referred to as the neutral orientation) the femur is impacted laterally along its frontal axis (Fy). Positive values of $\alpha$ and $\beta$ correspond respectively to posterior and medial orientations. For each fall impact orientations considered and for each subject in the above cohort femur, fall strength was predicted by Altai *et al.*^1^ using finite-element (FE) analysis (ANSYS Mechanical APDL, Ansys Inc., PA, USA). They used the full femur anatomy from the atlas (see above) to obtain the knee joint centre coordinates. Multi-point constraints (MPC) were used to establish at this location a rotational hinge centred at the pilot node around the axis transverse to the applied load, while all other degrees of freedom were fixed. At the greater trochanter, a non-linear surface-to-surface contact using augmented-Lagrange algorithm was employed between the surface of the greater trochanter and a static, rigid plate. A 500 N quasi-static load was applied and principal strains were computed on all proximal femur surface nodes except those close to where the boundary and contact constraints were applied. Nodal strains were averaged in 3 mm radius circular patches and the lowest minimum principal strain and the highest maximum principal strain were obtained. Altai *et al.*^1^ assumed that these peak strains scaled linearly with applied load. Bone strength was computed as the multiple of applied load that leads the peak strain with the larger absolute value to exceed a critical strain limit.^2^ They reported excellent fracture classification accuracy (AUC = 0.82)—for the cohort mentioned in the previous section—when the minimum patient-specific strength across all impact orientations (denoted Minimum fall strength or MFS) was used as the classifier. This demonstrates the pipeline’s credibility *in vivo*.^1^ This pipeline has been shown to achieve *ex vivo* accuracies of 7 and 15% for predicted strains and strength respectively.^28,29^

Note that Altai *et al.*^1^ reported results for a set of 28 impact orientations per subject (the full result dataset is freely available, see data URL in that paper). The authors of that study computed bone strength for five additional orientations per subject (total 33 orientations, see Supplementary Material Table S1 in the present manuscript) following the pipeline described above (personal communication). These orientations were always far away from the MFS orientation. As results from all 33 orientations were used in B18, these continue to be used in the present study as well. Indeed, no new FE analysis was conducted in the present study as the procedure for obtaining orientation-specific femur strength $S$ is identical to that in B18 (Fig. 1).

In B18 the body–floor impact and ground–skeleton force-transfer models were used synergically to estimate the impact load acting on the femur. In the body–floor impact model an inverted pendulum abstraction of the body during a fall was adopted to calculate the peak impact force exchanged with the ground given the height and weight of the patient. Then, the ground–skeleton force-transfer model considered the presence of damping effects such as flooring elements and active soft tissues (${\eta }_{\mathrm{I}}$ coefficient), as well as passive soft tissues (${\eta }_{\mathrm{ST}}$ coefficient), to determine the impact force transferred to the skeleton. In B18, the dependence of ${\eta }_{ST}$ on impact orientation was neglected, and ${\eta }_{\mathrm{ST}}$ was estimated from a population-based regression relation^24^ ${\eta }_{\mathrm{ST}}=0.0986\cdot STT0$. STT0 corresponds to an impact along the neutral hip orientation and was estimated from a regression relationship with the subject’s BMI.^4,19^ This regression relation is based on studies where STT0 was quantified from whole-body DXA images.^5,27^ These studies report a 1.5 mm inter-observer precision in the DXA-based measurement of STT0, and an 11.1 mm standard error in the regression relationship based on BMI.

In B18 ARF0 i.e. the absolute risk of fracture for each patient was computed by orchestrating the three afore-mentioned components models. An individual fall event was said to lead to a fracture ($\chi =1$) if the attenuated impact force magnitude ($F$) exceeded bone strength ($S$) along the impact orientation specified by the fall; otherwise, the variable $\chi$ was set to 0. To obtain the probability $P$ that an arbitrary fall would lead to a fracture, multiple fall scenarios were simulated and the variable $\chi$ was integrated over these scenarios. Gauss quadrature was used to integrate over the impact orientations and Monte-Carlo (MC) integration was used to integrate over all variables related to body–floor impact and ground–skeleton force-transfer models (except patient-specific variables body mass and body height). Each orientation was assumed to be equally likely and truncated symmetric Gaussian distributions were defined for the MC variables. These distributions were parameterised based on studies reported in the literature (details can be found in B18) and as such were not subject-specific. Inverse-transformed Latin hypercube sampling was used to generate samples for MC integration. The probability $P$ was used in conjunction with the annual fall rate to finally obtain the absolute current risk of hip fracture or ARF0.

The above baseline model prediction is henceforth referred to as ARF0_STT0-DXA-BMI_. Here, the subscript underscores that: (i) STT at the neutral (or ‘0’) orientation is used to approximate STT at every orientation; (ii) DXA is the source of the true STT0 measurement; and (iii) a regression model based on BMI is employed in lieu of the true measurement.

### ARF0 Using Subject- and Orientation-Specific Soft Tissue Thickness

**Figure 3:**
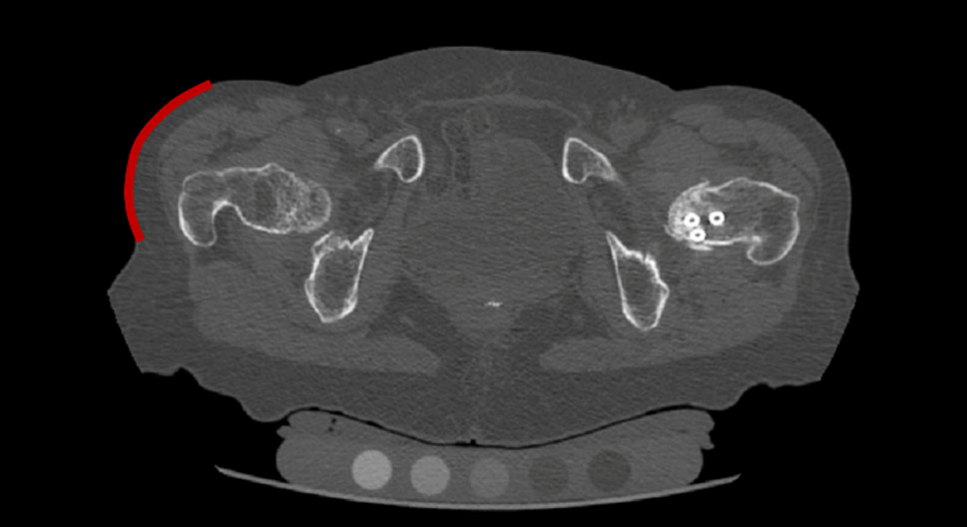
One CT slice with the pelvic surface highlighted in red. The surface was segmented in order to locate the end of the soft tissues surrounding the distal femur and measure the STT along the different orientations. The full soft-tissue profile is built by segmenting each CT slice in the image-set.

In the present study, the pelvic outer surface of each subject, i.e. the outer limit of the tissues overlying the greater trochanter (Fig. 3) was identified, segmented and exported as a polygonal surface using (Mimics 19.0, Materialise, Leuven, Belgium). The segmentation was carried out semi-automatically based on a fixed threshold.

The discretised geometry of the pelvic outer surface was aligned in the femur anatomical coordinate system, using the same transformation as applied to the femur surface to align it with its anatomical coordinate system. The ray starting from the femoral head centre and oriented along each impact orientation ($\alpha ,\beta$) was considered. The intersection(s) of this ray with the femoral surface and the intersection of this ray with the pelvic surface were determined. Of all possible intersections with the femoral surface, the one closest to the intersection with the pelvic surface was chosen and its distance was defined as $STT\left(\alpha ,\beta \right)$. The above analyses were performed in MATLAB version 2019b (MathWorks, Natick, USA). Note that all $STT\left(\alpha ,\beta \right)$ are measured from a single reconstructed CT image. Hence, these correspond to the subject maintaining a fixed (typically neutral) rotation and adduction of the hip.

In the present study, the ARF0 model of B18 is modified insofar as to include the orientation-dependent $STT\left(\alpha ,\beta \right)$ as input to the ground–skeleton force-transfer model models (Figure 1). Orientation-specific attenuation coefficients ${\eta }_{ST}\left(\alpha ,\beta \right)=0.0986\cdot STT\left(\alpha ,\beta \right)$ are derived using the same regression relation as B18 but considering orientation-dependent STT as the input. The current absolute risk of fracture determined in this fashion is referred to as ARF0_STT-CT_ to distinguish it from the baseline model. The subscript underscores that: (i) the full three-dimensional STT is used; and (ii) CT is the source of the true measurement.

### Statistical Analysis

Following the performance of Shapiro–Wilk normality test, Wilcoxon signed rank non-parametric test or parametric paired-sample t-test were used to determine statistical significance of pairwise differences between ARF0_STT0-DXA-BMI_ and ARF0_STT-CT_. Fracture status based on ARF0_STT-CT_ was predicted using a multivariate logistic regression model after adjusting for age, height and weight. Goodness of fit of the model was assessed using a Hosmer–Lemeshow test. Tests were carried out for the full cohort, and separately for the fracture and non-fracture groups. A Receiver Operating Characteristic (ROC) analysis was performed to assess the sensitivity and specificity of classifying fracture and non-fracture subjects using ARF0_STT-CT_. Unless stated otherwise, statistical significance is taken to be indicated by *p* < 0.001. The full set of results presented in this paper can be freely downloaded from the following URL: https://doi.org/10.15131/shef.data.15131631.

## Results

**Figure 4:**
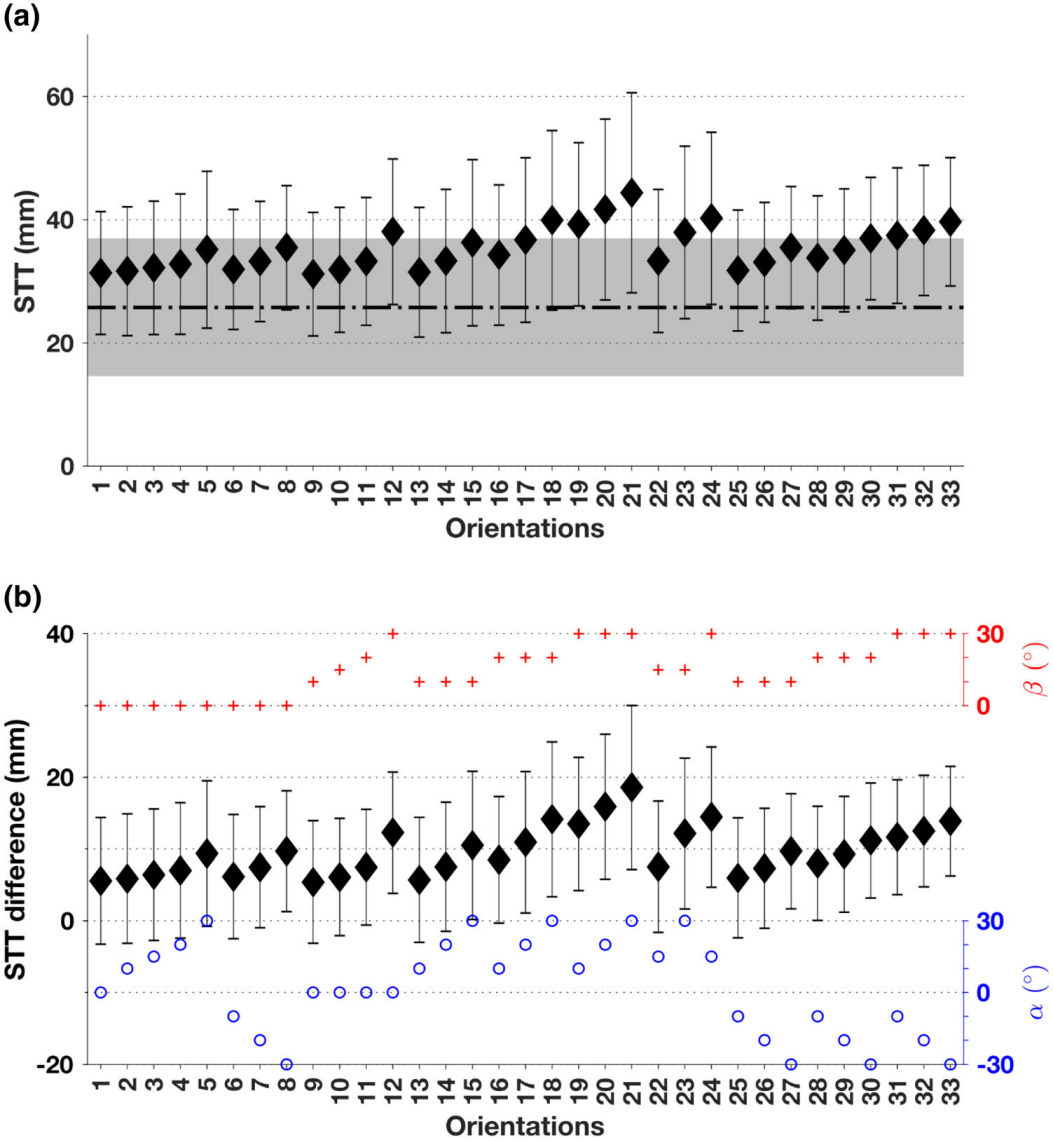
(A) Dependence of STT computed from CT on impact orientation: filled diamonds indicate mean and whiskers indicate standard deviation (SD) of STT across the cohort for fixed impact orientation. For reference, orientation-independent STT at greater trochanter (or STT0) estimated from BMI in Bhattacharya *et al.*^3^ is also shown: mean (26 mm, dashed-dotted line) and SD (11 mm, grey band). (B) Mean (filled diamonds) and SD (whiskers) of subject-specific differences between STT computed from CT and STT0 estimated from BMI. For reference, circle and plus symbols denoting respectively the impact orientation angles ($\alpha ,\beta$) are shown on the two split vertical axes on the right.

For the CT image resolution used in the present study, the geometry extraction process and the STT measurement has a precision of ~ 1 mm.^20^ Figure 4A gives an overview of the impact orientation dependent STT as determined from CT, together with STT0 estimated from BMI. Average STT at the neutral orientation (orientation label 1) determined from CT (31 mm) was found to be statistically significantly larger than that estimated from BMI (26 mm). Average STT increases further as one rotates away from the neutral orientation, whether along the posterior (labels 2–5), anterior (labels 6–8) or medial (labels 9–12) loading orientations, with the medial orientations registering the steepest increase in average STT per degree of rotation. These trends are maintained when orientations are combined (labels 13–33). Thus, on average, STT determined from CT at any impact orientation is always higher that STT0 estimated from BMI. The highest average STT determined from CT (44 mm) occurs at the simultaneously posterior- and medial-most impact orientation 21 ($\alpha ={30}^{\circ },\beta ={30}^{\circ }$).

Variation in STT across subjects, expressed in standard deviations (SD), was the smallest (9.7 mm) and the largest (16 mm) at impact orientations 26 ($\alpha ={-20}^{\circ },\beta ={10}^{\circ }$) and 21 ($\alpha ={30}^{\circ },\beta ={30}^{\circ }$) respectively. The SD of STT is, on average, 11 mm at a given orientation (across subjects) and 4.9 mm for a given subject (across orientations).

The larger variability of STT across subjects can potentially be explained by BMI.^4^ Indeed, additional regression analyses (*k*-fold cross-validated, *k *= 17, Supplementary Material Tables S2 and S3) showed that at each orientation BMI was statistically significantly correlated to the $STT \left(\alpha ,\beta \right)$ (*R* = 0.65–0.75). Subject-specific differences between $STT(\alpha ,\beta )$ determined from CT and STT0 estimated from BMI ranged between − 24 mm and 43 mm across the cohort and impact orientations. However, the median difference at any impact orientation was statistically significantly greater than zero (Fig. 4B).

The largest variation in STT across subjects can be detected at impact orientation 21, where the preferential occurrence of within-subject highest STT values (56 out of 94 subjects) is observed. The simultaneously anterior- and medial-most impact orientation, labelled 33 ($\alpha ={-30}^{\circ },\beta ={30}^{\circ }$), also accounts for a large proportion of within-subject highest STT values (29 subjects). Subject-specific lowest STT values did not show any preference for a particular impact orientation, with at most 12 subjects having the same impact orientation where their STT was the lowest. The average STT of a subject was statistically significantly correlated with BMI (*R* = 0.73), increasing by 1.7 mm for every 1 kg/m^2^ increase in BMI. Within-subject variation in STT, expressed in SD, ranged from 1.8 mm to 10 mm. This variation was statistically significantly and positively correlated with BMI. When normalised to the subject’s average STT, the variation (expressed in percentage) ranged from 5.1 to 32% but did not demonstrate a statistically significant correlation with BMI.

In the ARF0 modelling pipeline, the observable variable that is affected most immediately by STT is the attenuated impact force $F$ (Fig. 1). This provides a route for validation of the ground–skeleton force-transfer model. In B18, input variables of the body–floor impact (validated separately) and ground–skeleton force-transfer models (including body mass and body height) were sampled from their respective physiological distributions. The model was executed to obtain the corresponding distribution of $F$. The same approach is used in the present study as the body–floor impact model is identical to that in B18 and was already validated there. For the ground–skeleton force-transfer model of the present study the distribution of $F$ (mean 2.32 N, SD 1.07 N) overlaps with ranges reported in the literature: 0.475–2.5 kN (experiments of Laing and Robinovitch^10^) and 1.23–5.57 kN (model predictions of Lo and Ashton-Miller^14^). Thus, the model is considered validated.

As measured STT values are on average larger than those estimated from BMI, it is expected that ARF0_STT-CT_ values will be lower than ARF0_STT0-DXA-BMI_. Median and average ARF0_STT-CT_ values resulted equal to, respectively: whole cohort, 23 and 28%; non-fracture group, 15 and 16%; fracture group, 42 and 41%. The median subject-specific decrease, from ARF0_STT0-DXA-BMI_ to ARF0_STT-CT_, was found to be statistically significant for the whole cohort (8.8 percentage points or pp), as well as for the fracture (6.7 pp) and non-fracture groups separately (10 pp). ARF0_STT0-DXA-BMI_ and ARF0_STT-CT_ were found to be strongly correlated (statistically significant correlation *R* = 0.89).

**Figure 5:**
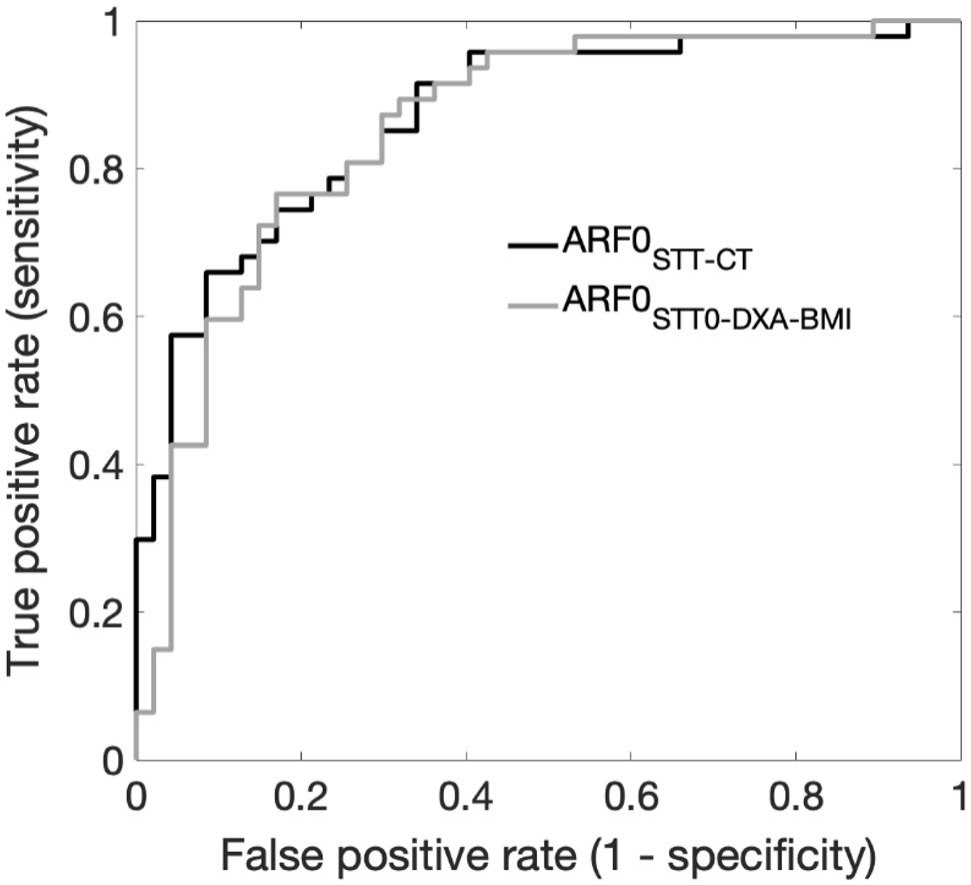
ROC curves for the fracture classification in the postmenopausal cohort using ARF0_STT-CT_ (black, AUC = 0.87) and ARF0_STT0-DXA-BMI_ (grey, AUC = 0.85).

Hosmer–Lemeshow test showed no evidence of poor fit when using multivariate logistic regression models for fracture status prediction based on ARF0_STT-CT_ (*p* = 0.36). Height-, weight and age-adjusted regression analyses showed that ARF0_STT-CT_ and ARF0_STT0-DXA-BMI_ remained significantly associated with the fracture status. An increase of ARF0_STT-CT_ and ARF0_STT0-DXA-BMI_ by one SD, while other predictive variables are held fixed, increase the odds of undergoing versus not undergoing a fracture by factors of 6.1 and 9.1 respectively. Figure 5 shows performance curves from ROC analyses. The most optimal classification occurred at ARF0_STT-CT_ = 21% threshold, with 79% sensitivity (95% CI 64–89%) and 79% specificity (95% CI 65–89%). The Area Under the ROC Curve (AUC) of 0.87 (95% CI 0.78–0.93) obtained using ARF0_STT-CT_ was larger (but not statistically significantly) than the AUC of 0.85 (95% CI 0.76–0.92) obtained using ARF0_STT0-DXA-BMI_.

## Discussion

The present study carried out an orientation- (i.e. three-dimensional) and subject-specific assessment of STT from CT scans. It aimed to investigate whether hip fracture classification accuracy improves significantly when a multiscale model of current absolute risk of hip fracture ARF0_STT0-DXA-BMI_ that was developed in a previous study^3^ was enhance using orientation- and subject-specific STT.

The precision of three-dimensional STT measurement using CT was found similar to that using whole-body DXA.^4^ Note that, in B18 and in the present study, true DXA measurements of STT0 were unavailable for comparison. In B18, STT0 was estimated from a regression relation between BMI and STT0 measured using whole body DXA, which on average was 5 mm lower than STT0 measured using CT. This is most likely a random effect, because Nielson *et al.*^19^ found that STT0 measurement using CT underestimated by 5 mm STT0 measured using whole body DXA (not BMI-based regression of the same). The opposite sense of the differences between CT and DXA, which are both smaller in magnitude than the uncertainty in the regression relation (11 mm) suggest that the difference of 5 mm reported here is a random and not systematic effect. Furthermore, it has no effect on classification accuracy as expected. Adding 5 mm to the regression relation between BMI and STT0 and carrying the change forward to estimate ${\eta }_{\mathrm{ST}}$ and ARF0_STT0-DXA-BMI+5_ led to no difference in fracture classification accuracy (AUC=0.85) relative to ARF0_STT0-DXA-BMI_.

The use of regression relationships as surrogates of subject-specific measurements can lower the precision of model input but also lower the cost of potential clinical pathways involving ARF0. The regression of CT-based three-dimensional $STT \left(\alpha ,\beta \right)$ on BMI can be used to recompute a fracture risk; henceforth, this is denoted by ARF0_STT-CT-BMI_. Here, STT in the subscript (replacing STT0) highlights the use of orientation-specific regression relationships. The accuracy of fracture classification using ARF0_STT-CT-BMI_ (AUC = 0.84; 95% CI 0.75–0.91) is poorer than that of ARF0_STT0-DXA-BMI_. Thus personalisation of STT (subject-specific measurement) is more important for fracture classification accuracy than characterising its three-dimensionality. This inference is further supported when three-dimensionality is suppressed in favour of using STT0 measurements from CT to compute a new risk value denoted by ARF0_STT0-CT_. It achieves an AUC (0.86; 95% CI: 0.77–0.92) between that of ARF0_STT0-DXA-BMI_ and ARF0_STT-CT_ when classifying fracture status in the cohort.

**Figure 6:**
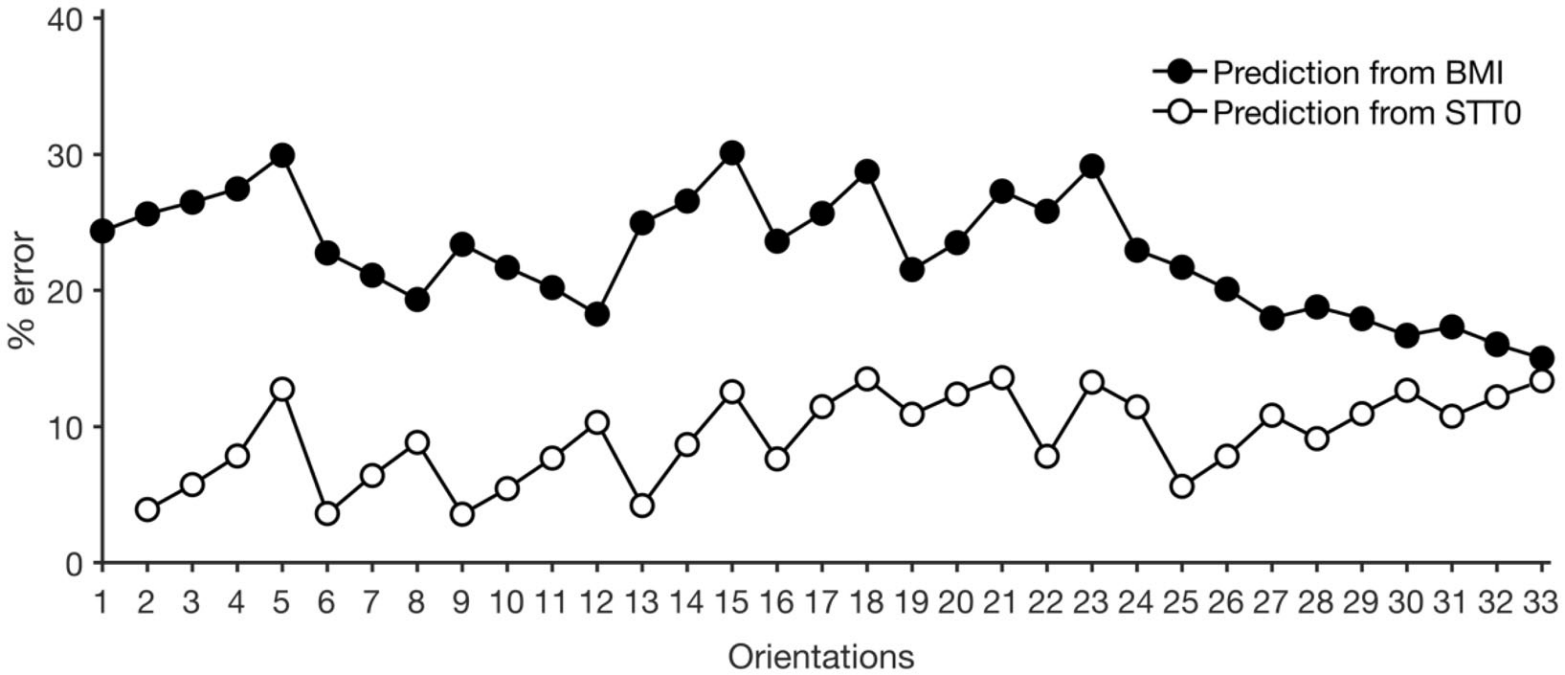
Mean absolute percentage error made in using BMI and STT0 based regressions to estimate STT at each orientation. Note that the error corresponding to first orientation, i.e. STT0, is not shown when STT0 is the predictor.

Using orientation-specific regressions of $STT \left(\alpha ,\beta \right)$ on STT0 measured by CT to compute fracture risk (denoted ARF0_STT-CT-STT0_) achieves an AUC of 0.87 (95% CI: 0.78–0.93) when classifying fracture status. This is higher than that of ARF0_STT0-CT_ and indistinguishable from that of ARF0_STT-CT_. ARF0_STT-CT-STT0_ achieves an optimal balance between imprecision due to use of regression relationships and accuracy of fracture classification. This is expected because, compared to BMI, STT0 is more strongly correlated to $STT \left(\alpha ,\beta \right)$ at each orientation (*R* = 0.78–0.99; Supplementary Material Tables S2 and S3). Here, the same *k*-fold cross-validated regression analysis described previously was used. Indeed, Fig. 6 shows that the mean absolute errors in predicting $STT\left(\alpha ,\beta \right)$ are considerably lower when using STT0 compared to BMI.

Although the differences in AUC reported here are quite small (0.84–0.87), when used to classify fracture status (or stratify the risk) in tens of thousands of subjects, these can have a substantial impact on net healthcare outcomes. It would be of interest to quantify in future studies the cost-effectiveness of using ARF0_STT-CT_ for the clinical management of hip fractures. The additional costs associated to the ARF0_STT-CT_ (AUC = 0.87) and ARF0_STT0-DXA-BMI_ (AUC = 0.85) models are negligible compared to the minimum fall strength (MFS) model predicted using CT-based FE (AUC = 0.82).^1^ This is because the component models in both ARF0 models—of body–floor impact and ground–skeleton force-transfer—require fully-automated computations including an inexpensive MC integration, which incur negligible costs compared to the multiple FE simulations needed in the MFS model. The effort to segment the skin/air boundary (needed for ARF0_STT-CT_) is negligible compared to that required to segment the femur boundary (needed for MFS). A recent study estimated that 23 hip fractures per 10000 person-years could be avoided using the MFS model relative to the standard-of-care approach using DXA aBMD (AUC = 0.75).^31^ Given that with respect to MFS, ARF0_STT-CT_ has higher accuracy at negligible additional cost, a detailed cost-effectiveness analysis is very appealing.

Our study has several limitations. CT images are acquired in a supine position where the soft tissues spread laterally and influence STT measurements. This influence has been noted previously in studies using whole body DXA.^4,19,27^ Lafleur *et al.*^9^ used US measurements to show that compared to a standing position, lying supine leads to overestimation of STT (29% on average) for all hip rotations. However, the body position that serves as the true reference is the one used in a previous study^24^ based on which the regression relation between STT and impact force attenuation was developed. There, trochanteric soft tissues were excised from cadavers prior to STT measurement. It is not straightforward to infer whether STT measured in that *ex vivo* study differs systematically or randomly from STT measured *in vivo* using CT in the present study.

Another methodological limitation due to CT imaging is that images are obtained in the neutral hip orientation only. In general, the volume of soft tissue increases towards the posterior and superior aspects of the greater trochanter. This explains why the top six impact orientations where the highest STT was most frequently encountered corresponded to posterior and medial angles ($\alpha \ge {10}^{\circ },\beta \ge {20}^{\circ }$). However, STT measured from CT images at these posterior and medial orientations approximates the STT available to attenuate falls with internally rotated and adducted hips, respectively. The direction of error in the above approximation can be inferred considering that when the femur moves with respect to the pelvis it does not “carry” the soft tissues with it. This implies that adduction and/or internal rotation cause the greater trochanter to move to a region of relatively less STT; the opposite is the case for an external rotation. Unlike CT, US measurements can be taken at multiple hip rotations on the same subject. Lafleur *et al.*^9^ found STT to be the lowest for the 25º internally rotated hip (posterolateral impact), compared to neutral and 25º externally rotated hips (anterolateral impact). Thus, STT measurements reported at posterior and anterior impact directions in the present study contrast with those measured by Lafleur *et al.*^9^ Yet, when Lim *et al.*^13^ used US to measure STT at the instant of impact, they found STT to be 8% greater in posterolateral than in anterolateral orientations (leading to 62% higher energy absorption in the former). While these contrasting results motivate further research to reconcile methodological dissimilarities, the present study found orientation-specificity to have a minor influence on fracture classification accuracy. This agrees with the finding of Lafleur *et al.* that hip rotations did not lead to clinically relevant differences in STT.^9^

In the abovementioned study by Robinovitch *et al.*,^24^ a synthetic femur covered with the excised soft tissue was impacted in the neutral orientation. Hence, the regression relation to predict impact force attenuation corresponds to that impact orientation only. A limitation of the present study is that the same regression relationship is assumed to satisfactorily estimate attenuation coefficients irrespective of orientation. Testing the validity of this assumption and potentially developing impact orientation specific regression relationships will require conducting new cadaver experiments which are outside the scope of the present study.

Finally, the research hypothesis of the present study limits the contexts in which the ARF0 model can be applied. Here, the hypothesis is that classification accuracy is improved by personalised 3D assessment of STT. Classification accuracy inherently depends on the subject-specificity of the model. Hence, the component models of ARF0 exclude mechanical knowledge (i.e. model sophistication) that required subject-specific information not available in the Sheffield cohort (e.g. MRI data). The limitation is that this approach needs to be reevaluated if the hypothesis were different. For example, taking a frequentist view leads to the expectation that in a sufficiently large cohort (or a virtual population) the average ARF0 across subjects predicts the fraction of fractured subjects. When testing this hypothesis, the numerical value of average ARF0 can be compared to an empirically observable quantity (fracture incidence) and subject-specificity of ARF0 (who has/has not fractured) is less important.

In conclusion, this study considered improving STT assessment by increasing subject- and/or orientation-specificity. Overall, using a more precise assessment of STT in computing ARF0 improves hip fracture classification accuracy in a cohort of British postmenopausal women. Increasing both subject- and orientation-specificity leads to the highest improvement in classification accuracy. The improvement diminishes if only subject-specificity is increased and disappears altogether if only orientation-specificity is increased. Compared to a CT-FE based MFS model, hip fracture classification using an ARF0 model informed by subject- and orientation-specific STT incurs negligible additional computational costs and substantially improves accuracy. This motivates the need to quantify its cost-effectiveness in hip fracture management within a clinical setting in future studies.

## Supplementary Information

Below is the link to the electronic supplementary material.

Supplementary file1 (PDF 230 kb)
