# Supplementary material for: Personalised 3D Assessment of Trochanteric Soft Tissues Improves HIP Fracture Classification Accuracy
Source: Ann Biomed Eng. 2022 Feb 1;50(3):303–13. doi: 10.1007/s10439-022-02924-1 (PMC8847196; doi:10.1007/s10439-022-02924-1)
Supplement: Supplementary file 1 — Supplementary file1 (PDF 230 kb) [file 10439_2022_2924_MOESM1_ESM.pdf]

Table S1 Combinations of α and β values defining each fall impact orientation. The angle α refers to rotation with respect to the anatomical longitudinal axis, with positive and negative values indicating posterior and anterior impact orientations respectively; β refers to rotation with respect to the sagittal axis, with positive values indicating an adducted hip.

Orientation	α(°)	β(°)	Orientation	α(°)	β(°)
**1**	0	0	**18**	30	20
**2**	10	0	**19**	10	30
**3**	15	0	**20**	20	30
**4**	20	0	**21**	30	30
**5**	30	0	**22**	15	15
**6**	-10	0	**23**	30	15
**7**	-20	0	**24**	15	30
**8**	-30	0	**25**	-10	10
**9**	0	10	**26**	-20	10
**10**	0	15	**27**	-30	10
**11**	0	20	**28**	-10	20
**12**	0	30	**29**	-20	20
**13**	10	10	**30**	-30	20
**14**	20	10	**31**	-10	30
**15**	30	10	**32**	-20	30
**16**	10	20	**33**	-30	30
**17**	20	20			

Table S2 Regression analyses coefficients for each orientation, as obtained from the 17-fold cross validation. Both mean values and standard deviations are shown.

Orientation	BMI-based Regression				STT0-based Regression			
	Intercept		Slope		Intercept		Slope	
	Mean	SD	Mean	SD	Mean	SD	Mean	SD
**1**	-3.32	1.26	1.37	0.05	-	-	-	-
**2**	-4.52	1.32	1.43	0.05	-0.81	0.13	1.04	0.00
**3**	-5.37	1.37	1.49	0.05	-1.05	0.17	1.06	0.01
**4**	-6.49	1.47	1.55	0.06	-1.57	0.24	1.10	0.01
**5**	-8.53	1.80	1.73	0.07	-1.24	0.39	1.16	0.01
**6**	-2.41	1.25	1.36	0.05	1.63	0.14	0.97	0.00
**7**	-2.02	1.24	1.39	0.05	3.63	0.25	0.94	0.01
**8**	-1.43	1.30	1.46	0.05	6.25	0.34	0.93	0.01
**9**	-4.96	1.20	1.43	0.05	0.17	0.13	0.99	0.01
**10**	-6.28	1.17	1.51	0.05	0.94	0.17	0.99	0.01
**11**	-6.46	1.16	1.57	0.05	2.27	0.24	0.99	0.01
**12**	-7.72	1.14	1.81	0.05	4.20	0.34	1.08	0.01
**13**	-6.37	1.26	1.50	0.05	-1.06	0.16	1.04	0.01
**14**	-9.19	1.46	1.68	0.05	-1.21	0.27	1.10	0.01
**15**	-11.20	1.76	1.88	0.07	-2.65	0.45	1.24	0.02
**16**	-7.61	1.30	1.66	0.05	-0.09	0.35	1.10	0.01
**17**	-12.25	1.36	1.94	0.05	-2.44	0.41	1.25	0.01
**18**	-12.41	1.67	2.07	0.06	-1.74	0.51	1.33	0.02
**19**	-11.51	1.28	2.01	0.05	0.48	0.37	1.24	0.01
**20**	-14.78	1.45	2.23	0.06	-1.10	0.37	1.36	0.01
**21**	-16.54	1.77	2.41	0.07	-2.26	0.52	1.49	0.02
**22**	-8.45	1.34	1.65	0.05	-1.65	0.27	1.12	0.01
**23**	-11.95	1.62	1.97	0.06	-2.46	0.47	1.29	0.02
**24**	-13.04	1.43	2.11	0.06	-0.65	0.37	1.30	0.01
**25**	-4.05	1.21	1.42	0.05	1.95	0.16	0.95	0.01
**26**	-2.39	1.28	1.40	0.05	4.43	0.28	0.91	0.01
**27**	-2.22	1.19	1.49	0.04	8.32	0.30	0.87	0.01
**28**	-4.96	1.18	1.53	0.05	4.49	0.22	0.93	0.01
**29**	-2.69	1.20	1.49	0.05	7.45	0.31	0.88	0.01
**30**	-0.81	1.24	1.49	0.05	11.80	0.35	0.80	0.01
**31**	-5.71	1.21	1.71	0.05	6.54	0.30	0.99	0.01
**32**	-3.50	1.28	1.65	0.05	10.28	0.35	0.89	0.01
**33**	-1.95	1.34	1.65	0.05	13.93	0.35	0.82	0.01

Table S3 Regression analyses coefficients as obtained from the 5- and 10-fold cross validation procedures. Both mean values and standard deviations are shown.

O.	10-fold cross-validation							5-fold cross-validation							
	BMI-based Regression				STT0-based Regression			BMI-based Regression				STT0-based Regression			
	Intercept		Slope		Intercept		Slope	Intercept		Slope		Intercept		Slope	
	Mean	SD	Mean	SD	Mean	SD	Mean	Mean	SD	Mean	SD	Mean	SD	Mean	SD
**1**	-3.32	1.27	1.37	0.04	-	-	-	-3.41	0.96	1.37	0.05	-	-	-	-
**2**	-4.53	1.48	1.43	0.05	-0.81	0.19	1.04	-4.63	1.11	1.43	0.06	-0.8	0.29	1.04	0.01
**3**	-5.39	1.6	1.49	0.06	-1.05	0.24	1.06	-5.49	1.41	1.49	0.07	-1.04	0.49	1.06	0.01
**4**	-6.51	1.76	1.55	0.07	-1.57	0.32	1.1	-6.61	1.75	1.56	0.08	-1.55	0.75	1.1	0.02
**5**	-8.56	2.37	1.73	0.09	-1.23	0.5	1.16	-8.62	2.62	1.73	0.11	-1.2	1.15	1.16	0.03
**6**	-2.4	1.35	1.36	0.05	1.64	0.22	0.97	-2.48	0.65	1.36	0.04	1.63	0.37	0.97	0.01
**7**	-2.01	1.45	1.39	0.05	3.64	0.38	0.94	-2.06	0.68	1.4	0.03	3.62	0.57	0.94	0.02
**8**	-1.41	1.62	1.46	0.06	6.26	0.52	0.93	-1.45	0.83	1.46	0.04	6.24	0.59	0.93	0.02
**9**	-4.96	1.18	1.43	0.04	0.18	0.24	0.99	-5.02	1.02	1.43	0.06	0.19	0.16	0.99	0.01
**10**	-6.28	1.07	1.51	0.04	0.94	0.29	0.99	-6.3	1.09	1.51	0.06	0.96	0.21	0.99	0.01
**11**	-6.46	1.14	1.57	0.04	2.27	0.34	0.99	-6.49	0.95	1.57	0.05	2.29	0.18	0.99	0.01
**12**	-7.73	1.29	1.81	0.05	4.19	0.52	1.08	-7.72	1.3	1.81	0.06	4.23	0.44	1.08	0.02
**13**	-6.38	1.25	1.5	0.04	-1.06	0.19	1.04	-6.45	1.18	1.5	0.06	-1.04	0.36	1.04	0.01
**14**	-9.21	1.62	1.68	0.06	-1.2	0.23	1.1	-9.25	1.94	1.68	0.08	-1.18	0.77	1.1	0.02
**15**	-11.23	2.23	1.88	0.09	-2.65	0.39	1.24	-11.3	2.25	1.88	0.1	-2.63	1.2	1.24	0.03
**16**	-7.62	1.22	1.66	0.04	-0.09	0.37	1.1	-7.65	1.35	1.66	0.06	-0.07	0.68	1.1	0.02
**17**	-12.29	1.66	1.94	0.07	-2.45	0.47	1.25	-12.32	1.69	1.94	0.08	-2.42	0.9	1.25	0.02
**18**	-12.47	2.3	2.07	0.09	-1.75	0.53	1.33	-12.52	2.66	2.07	0.11	-1.71	1.48	1.33	0.04
**19**	-11.54	1.44	2.01	0.05	0.46	0.49	1.24	-11.53	1.56	2.01	0.07	0.5	0.84	1.24	0.03
**20**	-14.83	1.95	2.23	0.08	-1.11	0.38	1.37	-14.91	2.05	2.24	0.09	-1.08	1.06	1.36	0.03
**21**	-16.59	2.35	2.41	0.09	-2.28	0.51	1.49	-16.66	2.64	2.41	0.11	-2.23	1.58	1.49	0.04
**22**	-8.47	1.31	1.65	0.05	-1.65	0.3	1.12	-8.53	1.75	1.65	0.08	-1.63	0.68	1.11	0.02
**23**	-11.99	2.25	1.97	0.09	-2.46	0.43	1.29	-12.08	2.37	1.98	0.1	-2.43	1.26	1.29	0.04
**24**	-13.08	1.64	2.11	0.06	-0.66	0.42	1.31	-13.1	1.94	2.11	0.08	-0.62	1.01	1.3	0.03
**25**	-4.04	1.22	1.42	0.04	1.96	0.37	0.95	-4.08	0.69	1.42	0.04	1.96	0.23	0.95	0.01
**26**	-2.36	1.5	1.4	0.05	4.44	0.52	0.91	-2.4	0.93	1.4	0.05	4.45	0.49	0.91	0.02
**27**	-2.17	1.75	1.49	0.07	8.34	0.56	0.87	-2.15	1.14	1.49	0.05	8.32	0.5	0.87	0.01
**28**	-4.94	1.25	1.53	0.04	4.5	0.58	0.93	-4.96	0.61	1.53	0.04	4.51	0.41	0.93	0.02
**29**	-2.66	1.36	1.49	0.05	7.46	0.68	0.88	-2.67	0.62	1.49	0.04	7.47	0.55	0.88	0.02
**30**	-0.76	1.92	1.49	0.07	11.82	0.8	0.8	-0.78	0.78	1.49	0.04	11.82	0.87	0.8	0.03
**31**	-5.7	1.33	1.71	0.05	6.54	0.55	0.99	-5.71	0.97	1.71	0.05	6.57	0.26	0.98	0.01
**32**	-3.48	1.58	1.65	0.06	10.29	0.67	0.89	-3.54	1.18	1.65	0.06	10.32	0.55	0.89	0.02
**33**	-1.92	1.55	1.64	0.06	13.93	0.74	0.82	-1.94	1.08	1.64	0.06	13.96	0.62	0.82	0.03
